# Role of a Dual Glucose-Dependent Insulinotropic Peptide (GIP)/Glucagon-like Peptide-1 Receptor Agonist (Twincretin) in Glycemic Control: From Pathophysiology to Treatment

**DOI:** 10.3390/life12010029

**Published:** 2021-12-25

**Authors:** Maria Chiara Pelle, Michele Provenzano, Isabella Zaffina, Roberta Pujia, Federica Giofrè, Stefania Lucà, Michele Andreucci, Angela Sciacqua, Franco Arturi

**Affiliations:** 1 Department of Medical and Surgical Sciences, University “Magna Graecia” of Catanzaro, 88100 Catanzaro, Italy; mcpelle@unicz.it (M.C.P.); zaffina86@gmail.com (I.Z.); robertapuj@gmail.com (R.P.); federica.giofre@gmail.com (F.G.); lu.stef@tiscali.it (S.L.); sciacqua@unicz.it (A.S.); 2 Department of Health Sciences, University “Magna Graecia” of Catanzaro, 88100 Catanzaro, Italy; michiprov@hotmail.it (M.P.); andreucci@unicz.it (M.A.)

**Keywords:** hyperglycemia, GIP, twincretin, pathophysiology, therapeutics

## Abstract

Glucagon-like peptide-1 (GLP-1) and glucose-dependent insulinotropic polypeptide (GIP) are two gut hormones, defined incretins, responsible for the amplification of insulin secretion after oral glucose intake. Unlike GLP-1, GIP has little acute effect on insulin secretion and no effect on food intake; instead it seems that the GIP may be an obesity-promoting hormone. In patients with type2 diabetes mellitus (T2DM) some studies found a downregulation of GIP receptors on pancreatic β cells caused by hyperglycemic state, but the glucagonotropic effect persisted. Agonists of the receptor for the GLP-1 have proven successful for the treatment of diabetes, since they reduce the risk for cardiovascular and renal events, but the possible application of GIP as therapy for T2DM is discussed. Moreover, the latest evidence showed a synergetic effect when GIP was combined with GLP-1 in monomolecular co-agonists. In fact, compared with the separate infusion of each hormone, the combination increased both insulin response and glucagonostatic response. In accordance with theseconsiderations, a dual GIP/GLP-1receptor agonist, i.e., Tirzepatide, known as a “twincretin” had been developed. In the pre-clinical trials, as well as Phase 1–3 clinical trials, Tirzepatideshowedpotent glucose lowering and weight loss effects within an acceptable safety.

## 1. Introduction

The discovery of the incretin dates to 1929 due to the work of La Barre [1], but the true pioneers of the incretin concept were Bayliss and Moore 20 years earlier, when they hypothesized that the endocrine pancreas secretion was regulated by hormones contained in the gut extracts [2,3]. Incretin was forgotten for three decades until the discovery by McIntyre [4] that stated that intestines released some humoral substances, after glucose ingestion, that helped to promote β-cellular secretion of insulin, followed by the introduction by Unger and Eisentrout of the concept of “entero-insular axis” [5].

The two best studied incretins are glucagon-like peptide-1 (GLP-1) and glucose-dependent insulinotropic polypeptide (GIP). GLP-1 is a 30-amino acid peptide, secreted from intestinal L cells in response to meal ingestion [6,7]. GIP, originally isolated from porcine intestine, is a 42-amino-acid hormone secreted from K-cells of the upper small intestine [8,9].

Both GLP-1 and GIP are inactivated by DPP-4, resulting in a short half-life [10]. The GLP-1 receptor is expressed in the pancreas, gastrointestinal tract, kidney, heart, and brain [11]. The insulinotropic property, gluconostatic effect during hyperglycemia, and normoglycaemic state of GLP-1 have been well demonstrated. Moreover, this hormone has several extrapancreatic properties; in fact GLP-1 promotes satiety, reduces food intake, and slows gastric emptying [12].

In 2005, the FDA approved the first GLP-1 receptor agonists, exenatide, which followed with several studies demonstrating their efficacy and safety in T2DM subjects. In fact, this pharmacological class improved glucose homeostasis and reduced body weight [13], but interestingly, large cardiovascular outcome trials showed that GLP-1 agonists were not only well tolerated, but also reduced the risk of cardiovascular and renal outcomes in T2DM patients [14].

Although GIP was the first to be isolated, for many years its function was unclear. Unlike GLP-1, some studies demonstrated that the glucanotropic effects of GIP was present in the normoglycaemic and hypoglycaemic state and was unclear in the hyperglycaemic state [15].

During the chronic hyperglycaemic state, GIP receptors on pancreatic β cells are down regulated, but the insulinotropic effect can be restored by improving glycemic control [16].

Only in recent years has scientific research focused its interest on the synergistic action of GLP-1 and GIP. In vitro and in vivo preclinical studies have demonstrated that co-infusion of GLP-1 and GIP leads to improvement of glucose control and bodyweight regulation [17], compared with separate administration of each hormone. These findings have been confirmed in human studies.

Based on promising data, a dual GIP/GLP-1 receptor agonist, named “twincretin”, has been developed. There is emerging evidence on two molecules, NNC0090-2746 and LY3298176. The first molecule, developed by Novo Nordisk, is a fatty-acylated GIP/GLP-1 dual agonist, showed to improve glycated hemoglobin and to reduce bodyweight in patients with T2DM, compared to liraglutide in a Phase 2 clinical trial [18], (whereas no Phase 3 clinical trials are currently ongoing). The second molecule (developed by Eli Lilly and Company; Indianapolis, IN, USA), has passed Phase 1 and Phase 2 trials, and a Phase 3 clinical trial program (SURPASS Program) of this dual GIP/GLP-1 RA, named tirzepatide is ongoing [19], testing the long-term efficacy, safety, and cardiovascular outcomes of tirzepatide.

This review provides an overview regarding the effects of GIP/GLP-1 receptor agonist on the improvement of glucose control in T2DMpatients, as well as the emerging and relevant findings derived from preclinical and clinical studies focused on the Phase 3 clinical trial program of tirzepatide.

## 2. GIP Physiology: Similarities and Differences versus GLP-1

Glucose-dependent insulinotropic polypeptide (GIP) was the first gut hormone [10] to be isolated in 1970–1975 [20]. GIP and glucagon-like peptide-1 (GLP-1), the second intestinal hormones discovered, are called incretins [21], as they improve the insulin response after oral glucose administration, compared to that obtained following intravenous administration [1,22,23]. GIP is a 42-amino acid polypeptide secreted from enteroendocrine K-cells that are found in the small intestinal epithelium [24,25]. It originates from the post-translational processing of pre-pro-GIP and its structure is similar to secretin, glucagon, vasoactive intestinal peptide, and growth hormone-releasing factor [8]. K-cells express a variant of sodium-coupled glucose transporter-1 (SGLT-1), that works as a sensor for incretin secretion after oral ingestion of nutrients, such as glucose, amino acids, and long-chain fatty acids [26,27]. The half-life of GIP is 5 to 7 min [28], since it is rapidly inactivated by the ubiquitous enzyme dipeptidyl peptidase 4 (DPP-4) [29] that cuts alanine and proline residues in position 2 of the N-terminus in peptide chains [30]. GIP receptors (GIPRs) are found in several tissues, such as adipose tissue, bone, adrenal cortex, heart, pituitary, and cerebral cortex, hippocampus, and olfactory bulb [31], but their role at many of these sites is uncertain [20]. As previously mentioned, the incretin effect, for which following the ingestion of food the K-cells secrete the GIP, which by binding to its receptor located on the pancreatic β cells increases insulin secretion in a glucose-dependent manner. This mechanism includes the increase of cAMP, inhibition of K_ATP_ channels, increases in intracellular Ca^2+^, and stimulation of exocytosis [32,33]. In addition, GIP stimulates the transcription of β-cell insulin gene [34]. In the central nervous system, GIP could stimulate neural cell proliferation and modify behavior [35]. On adipose tissue, GIP has anabolic properties, activating fatty acid synthesis and reducing glucagon-mediated lipolysis [10]. Regarding bone, GIP induces proliferation of osteoblasts and inhibits osteoclastic bone resorption [36]. Finally, GIP decreases gastrin dependent acid secretion from the parietal cells of the stomach [37], but only at supraphysiologic doses [38].

**Figure 1:**
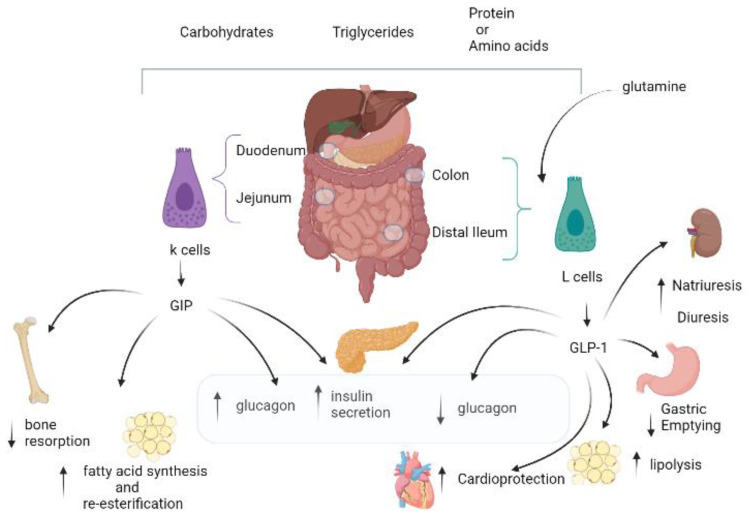
GIP and GLP-1: similarities and differences. GIP is secreted from K-cells of the proximal small intestine (duodenum and jejunum), while GLP-1 is secretedfrom L-cells of the small and large intestine (distal ileum and colon), following an introduction of carbohydrates, triglycerides, protein or amino acids. An important exception is glutamine, which is a specific stimulator for GLP-1. GIP and GLP-1determine insulin secretion in a glucose dependent manner. The actions of GLP-1 and GIP on glucagon secretion are differing, GLP-1 suppresses glucagon during hyperglycemia, but not at a normal fasting plasma glucose concentration, whereas GIP can stimulate glucagon secretion at fasting glycemic, during hypoglycemia, and hyperglycemia. Regarding adipose tissue, GLP-1 stimulates lipolysis, while GIP leads an accumulation of body fat.

Incretins have similar, but not identical, biological characteristics. First, the secretion of GIP and GLP-1 following the ingestion of nutrients is different. In fact, for the secretion of GIP their absorption is necessary, while for GLP1 their presence in the lumen would seem to be sufficient [39,40]. GIP is secreted from K-cells of the proximal small intestine (duodenum and jejunum) [8,41], while GLP-1 secretion from L-cells of the small and large intestine (distal ileum and colon) [42] following an introduction of carbohydrates, triglycerides, protein, or amino acids. An important exception is glutamine, which is a specific stimulator for GLP-1 [43]. It is important to underline that the GIP response does not depend on the quantity of the food ingested as on the composition [44] (Figure 1).

**Figure 2:**
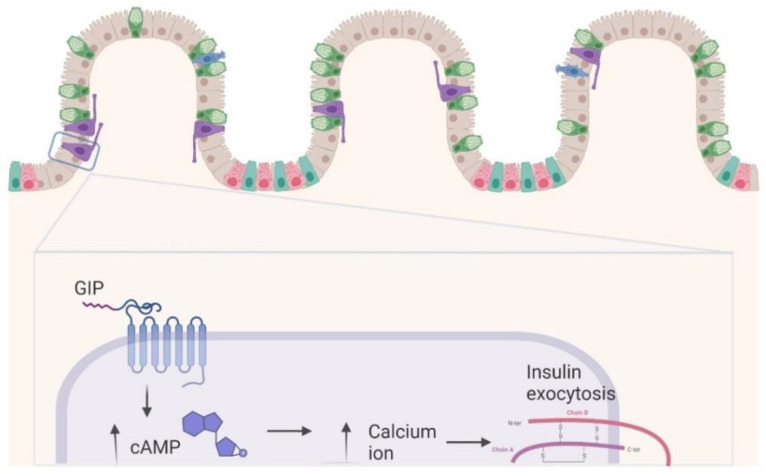
GIP receptors.GIP receptors (GIPRs) found in a variety of tissues are class-II G-protein coupled receptors whose mechanism involves the activation of adenylate cyclase/protein kinase A and cascades of phospholipase C/protein C so that binding of GIP to its receptor increases intracellular cAMP levels with a downstream increase in calcium ion concentration and insulin exocytosis; however, if they are not stimulated by the GIP they are internalized and recycled.

The half-life of GIP and bioactive GLP-1 is only a few minutes [45,46] since they are inactivated by DPP-4 [47]. Both GLP-1 and GIP receptors are expressed on pancreatic alpha and β-cells [48,49,50,51]. Moreover, GIP receptors are found on δ and PP cells [45]. GIP and GLP-1 exercise their effects by GIP and GLP-1 receptors [49,52,53,54,55,56,57,58], respectively, that are G-protein coupled receptors expressed on pancreatic β-cells. Their mechanism involves the activation of adenylate cyclase/protein kinase A and cascades of phospholipase C/protein C so that binding of GIP to its receptor increases intracellular cAMP levels with a downstream increase in calcium ion concentration and insulin exocytosis [29]. This determines insulin secretion in a glucose dependent manner [10,59]; however, if they are not stimulated by the GIP they are internalized and recycled [60,61] (Figure 2).

Moreover, they improve β-cell proliferation and survival [62]. The actions of GLP-1 and GIP on glucagon secretion are different, since GLP-1 suppresses glucagon [63] during hyperglycemia [64,65,66], but not at a normal fasting plasma glucose concentration [65]. GIP can stimulate glucagon secretion at fasting glycemic [67], during hypoglycemia, and hyperglycemia [64,68]. There is a close relationship between adipose tissue and GIP; fats strongly augment GIP secretion [69,70] and GIP levels are high in obese T2DM patients [71]. Although GLP1 stimulates lipolysis [10,71,72,73,74,75,76,77], GIP leads to the accumulation of body fat through fatty acid synthesis and re-esterification, augmentation of integration of fatty acids into triglycerides, increased synthesis of lipoprotein lipase, and reduction of lipolysis. GIP, and perhaps to a lesser extent GLP-1, which does not present clear evidence of bone metabolism, act on bone remodeling through the effects on osteoclasts and osteoblasts [43]. Incretins have been shown to indirectly affect hepatic and muscle metabolism, specifically through changes in circulating concentrations of insulin and glucagon [31,78,79,80]. Receptors for incretins found in the brain involved regulating feeding and energy expenditure. Furthermore, they are involved in synaptic plasticity [31,81], memory functions, and emotional responses, with the possibility of beneficial effects for a series of neurodegenerative disorders [81,82]. The effect of incretins on the gastrointestinal tract is opposite; GLP-1 slows gastric emptying [83,84,85], whereas this effect is not confirmed for GIP [86]. GIP and GLP-1 receptors are expressed in the cardiovascular system [33,79,80], where for the former the physiologic actions in these tissues are not known, while for the latter the beneficial action is known in terms of improving myocardial function and endothelial dysfunction, as well as increasing heart rate [10].

### The Dual GIP/GLP-1 Mechanisms

Although the GIP was the first to be isolated, for many years its function was unclear in the hyperglycaemic state; therefore, for this reason it was not used as a target for diabetic therapy, unlike GLP-1. Only recently scientific research has focused its interest on the synergistic action of GLP-1 and GIP, and as such several studies have been carried out and some are still ongoing.

**Figure 3:**
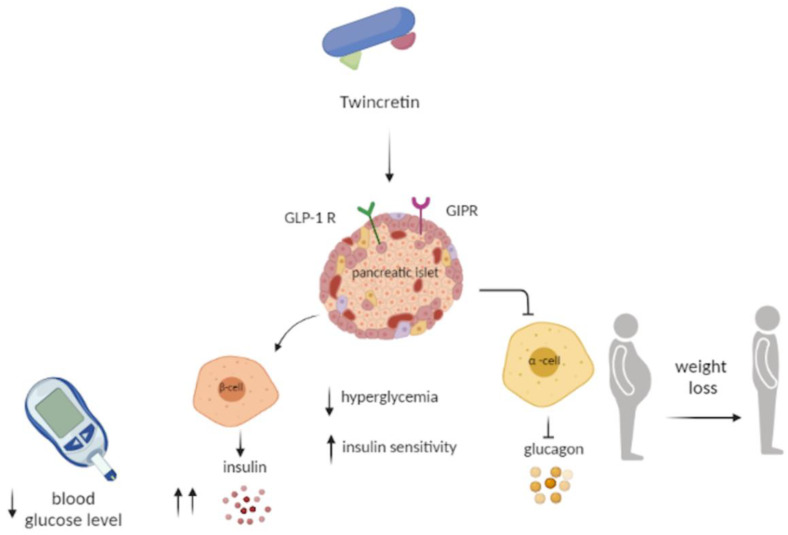
**The dual GIP/GLP-1 mechanisms.** GIP and GLP-1 receptors are both found in pancreatic β cells; the co-activation produces a more important gluconostatic effect and enhances insulin secretion than the single administration. Moreover, it could ameliorate food intake, body weight, and fat mass.

GIP and GLP-1 receptors are both found in pancreatic β cells. It is hypothesized that dual agonists can improve insulin secretion directly. Furthermore the reduction of the hyperglycemic state reduces the resistance to GIP [18,87]. Both receptors are present in the metabolic centers of the central nervous system, while only the GIP receptors are present in the adipose tissue, therefore their activation is assumed to be complementary to that of GLP-1 [88]. The co-activation can directly and indirectly improve insulin sensitivity, with an addictive effect. As will be discussed later, the combined infusion of GIP and GLP-1 produces a more important gluconostatic effect and enhanced insulin secretion than the single administration [89] (Figure 3).

The molecular mechanism of unimolecular incretins is not elucidated; however, they appear to correct insulin resistance due to adiposity and insulin secretion deficit. To understand whether the residual glucagon activity is a therapeutic target, fibroblast growth factor 21 (FGF21) and ketone bodies were measured as plasma markers indicative of chronic glucagon agonism. However, the administration of the dual incretin did not increase circulating FGF21 levels in vitro [90], while insulin secretion was greater than a single peptide. Animal studies demonstrated that co-stimulation could ameliorate food intake, body weight, and fat mass [90]. Furthermore, in human studies the main effect was increased insulin secretion [91] and inhibition glucagon one [89].

## 3. In Vitro Studies

Several in vitro studies have shown that the simultaneous administration of GIP and GLP-1 exert an additive effect on the processes that influenced the intracellular levels of cyclic adenosine monophasfate (cAMP) [92].

Gallwitz et al., (1993) proved an additive effect of GIP and GLP-1 on cyclic AMP production in RINm5F insulinoma cells (rat β-cells), demonstrating that the adenylate cyclase was influenced by signals produced from both receptors [93]. Later, Delmeire et al., (2004), explored the impact of GLP-1, GIP, or their combination on secretory function of the healthy rat β cell. This cell culture was performed for 24 h, added with either glucose alone or glucose with GLP-1, exendin-4, or GIP. They showed that GLP-1 acutely stimulated cellular cAMP production and enhanced glucose-stimulated insulin secretion, whereas prolonged contact with GLP-1 resulted in a time-dependent decrease in the cAMP. Furthermore, their data proved for the first time that a mixture of GLP-1 and GIP at physiological concentrations influenced the capacity to respond to later secretory stimuli independently of insulin content [94].

Lupi et al., (2010), tested the effects of acute (45 min) or prolonged (2 days) exposure to GLP-1 or GIP, alone or in combination on human pancreatic islets, that were obtained from non-diabetic and type 2 diabetic donors. β-cells were exposed acutely (45 min) with glucose, with or without the addition of GLP-1, GIP (in increasing doses), or a combination of GLP-1 and GIP. Furthermore, they were incubated for 48 h either with or without the addition of GLP-1, GIP, or a combination of both, followed by acute glucose stimulation. The authors demonstrated that GLP-1 and GIP have beneficial effects on insulin secretion both on-diabetic and type 2 diabetic human islets with acute or prolonged exposure to GLP-1 and GIP. In fact, pre-incubation with either GLP-1, GIP, or a combination of the two caused a 20–30% increase of insulin secretion. After prolonged exposure, the incretins administered together, therefore, exhibited synergistic action on both insulin synthesis and secretion, as well as effects on the genes associated with β-cell differentiation and survival, compared to the effects of individual peptides [95].

Furthermore, in vitro studies have documented that GIP may enhance the actions of GLP-1 by acting on the expression of both GLP-1R and GIPR. Incretin receptors belong to those coupled to G proteins, which function as monomers, heterodimers, or homodimers. Some [96,97] studies described that GLP-1R/GIPR heterodimer offered a reduced response to GLP-1 stimulation [98,99,100]. This conformation can be induced by GLP-1, while reversed by GIP [99].

## 4. Animal Studies

Animal studies did not provide unambiguous results. Certain studies demonstrated that a co-administration in ob/ob mice of GLP-1analogues, and GIP improved hypoglycemic, insulinotropic, and weight-reducing actions compared to the individual incretins [101,102,103,104,105,106,107].

Gault VA et al., (2011), evaluated the in vivo hypoglycemic effects of a Liraglutide and NAcGIP (Lys37Myr), a simple combination of Liraglutide plus N-AcGIP, or Lira-AcGIPpreparation (Lira-AcGIP) in healthy male Swiss TO mice and obese diabetic (ob/ob) mice in two phases. During the first, the acute (after 4 h from injection) glucose lowering and insulinotropic effects of incretins were evaluated on Swiss TO mice. Liraglutide and N-AcGIP established an important glycemic control, but the association of Liraglutide plus N-AcGIPdid not present a significant effect when compared to Liraglutide or N-AcGIP administered alone. Instead, the Lira-AcGIP preparation demonstrated an enhanced glycemic control compared to Liraglutide, N-AcGIP, and the simple combination of Liraglutide and N-AcGIP (17–45% reduction; *p* < 0.05–*p* < 0.001). Next, the second subchronic phase of in vivo study was conducted on obese diabetic ob/ob mice, during which Lira-AcGIP, Liraglutide, or N-AcGIP were administered once daily over 21 days. Liraglutide and Lira-AcGIP significantly reduced food intake, but only Lira-AcGIP reduced body weight [101].

Pathak NM et al., (2018) [105], compared the metabolic effects of the peptide, N-ac(D-Ala2)GIP/GLP-1-exe, a 56-amino acid hybrid peptide containing amino acids that allow it to bind to GIP and GLP-1 receptors, and with characteristics of exendin-4, known for enhanced metabolic stability and reduced clearance with respect to (D-Ala2) GIP, or exenin-4 therapy alone or in combination, administered twice daily.

Initially, acute effects of N-ac(D-Ala2) GIP/GLP-1-exe on glucose and insulin concentrations were evaluated demonstrating an evident 4 (*p* < 0.001) and 8 (*p* < 0.05) hours post-injection benefit on reducing serum glucose and improving insulin secretory compared to controls. Next, they proceeded to evaluate metabolic effects on high-fat-fed (HFF) mice of chronic treatment with N-ac(D-Ala2) GIP/GLP-1-exe, exendin-4 alone, and in combination with (D-Ala2)GIP, administrating twice daily over 28 days. This demonstrated a reduction of body weight, related with the loss of total body fat, while no change in lean body mass was documented, improving HbA1c, non-fasting blood glucose levels, and lipid profile, most likely due to enhanced insulin and β-cell insulin sensitivity when compared to the control group (saline). The hybrid peptide was able to act on the memory impairment associated with obesity-diabetes by improving it. Notably, the results of the second phase of this study obtained N-ac(D-Ala2) GIP/GLP-1-exe were analogous to (D-Ala2)GIP or exenin-4 therapy alone or in combination [106].

Furthermore, other studies demonstrated that the incretins together improved energy consumption and food intake in mice [101,108,109].

Irwin et al., (2007), studied the effects ofexendin-4 therapy administered once daily compared to saline for 12 days on a total of 14 HFF mice, followed by a further 12 days during which N-AcGIP was added. They demonstrated that exendin-4 caused a significant (*p* < 0.05) reduction in food intake during days 1 and 3 of the first phase of this study, while N-AcGIP had no effect on food intake when administered alone. Instead, the combined administration of GIP and GLP-1R agonist resulted in an increase in weight loss compared to GLP1R agonist alone, but no additional benefits in regulating glucose homeostasis [109].

However, other studies have shown that their simultaneous administration did not determine benefits when compared to the intake of incretins alone [110,111,112].

Irwin N. et al., (2007), studied the effects of sub-chronic (14 days) intra-peritoneal administration of N-AcGIP, exendin(1–39)amide, or the combination of both peptides or glucose in adult ob/ob mice. Ob/ob mice received intra-peritoneal injection of glucose alone (18mmol/kg body weight) or in combination with GIP, GLP-1, N-AcGIP, or exendin(1–39)amide. N-AcGIP alone or in combination with exendin(1-39)amide significantly decreased non-fasting plasma glucose HbA1c( *p* < 0.05) and improved glucose tolerance, but no significant effect on food intake or body weight between these groups was identified [110].

Killion et al., (2018), developed a mouse anti-murine monoclonal GIPR antibody (muGIPR-Ab) and an anti-human antagonist antibody(hGIPR-Ab). They treated GIPR knockout mice protected against diet induced obesity (DIO) with muGIPR-Ab or non-neutralizing GIPR antibody (CTL-Ab) for 45 days, resulting in a greater reduction in body weight of the muGIPR-Ab group, compared to control group. Furthermore, fasting blood glucose and serum insulin were reduced in the muGIPR-Ab treated group when compared to the control, while no enhancement in glucose tolerance was documented. Further, muGIPR-Ab, liraglutide, or a combination of both were administered to DIO mice, reaching a 23.5% of weight loss in the combination group. This last group achieved a reduction in food intake, as well as in the group with liraglutide alone. Next, they administered hGIPR-Ab, dulaglutide, a combination of both, or vehicle on nonhuman primates (NHPs) obtaining in the combination group a greater body weight reduction and a reduction of food intake, when compared to single treatments [113].

The rationale for these conflicting results could be due to the dose or desensitization of GLP-1R in the ob/ob mice used in the studies [110,114].

Finally, in these in vivo studies, the role of glucagon remains obscure, since mostly the levels have not been measured [110].

## 5. In Human Studies

To investigate individual and combined contributions of GLP-1 and GIP, Nuck et al., (1993), [91] administered both molecules in seven healthy volunteers, alone and combined, during an IV glucose infusion “isoglycemic” after an oral glucose tolerance test (50 g/400 mL). The β-cell response after administration of glucose IV alone and after single infusions of GLP-1 (7-36) amide (0.3 pmol/kg/min) and GIP (1 pmol/kg/min) was similar. Instead, when the two hormones were infused together, insulin secretion increased, thus demonstrating an additive effect. In another study, to establish the insulinotropic effect of two hormones, Elhai et al. [89] infused GLP-1 (7-37) amide and GIP into healthy subjects, as well as another group with diabetes mellitus. Different from GIP, during euglycemia, in the healthy subjects GLP-1 (7-37) stimulated insulin release. During hyperglycemia, induced with a clamp, insulin secretion increased when the hormones were co-infused, compared to the infusion of individual molecules. Moreover, incretins co-infusion induced a significant glucagonostatic effect, compared to separately infusion of glucose infusion alone.

In another study, a combination of synthetic GIP, synthetic GLP-1 (7-36) amide, and synthetic human glucagon-like peptide-2 (GLP-2) infused into ten subjects with diabetes, during an IV glucose infusion matching an OGTT, glucagon, and insulin secretion were measured. Mean body mass index (BMI) was 33.2 kg/m2 and mean HbA1c level was 6.8%; nine subjects were treated with metformin, or one metformin combined with sulphonylurea. In this study, GLP-1 infusion suppressed glucagon secretion, while GIP alone promoted glucagon response; when these hormones were administered together glucagon did not change. Insulin secretion increased during co-infusion, compared with GIP alone or GLP-2 alone, but not with GLP-1 alone; this result highlighted the lack of GIP stimulus on insulin release in patients with diabetes. Moreover, this study emphasized that GIP increased the inappropriate glucagon secretion after orally ingested glucose in subjects with T2DM [115].

Dousi et al., (2009) [116] included in their study six obese male patients with diet controlled T2DM (HbA1c < 8%) and six healthy lean male subjects. All participants received an infusion of synthetic human GIP (2 pmol/kg/min) combined with GLP-1 (1 pmol/kg/min) for 4 h. After which, co-infusion insulin was detected. In healthy subjects, insulin increased after co-infusion compared with GLP-1, GIP, or placebo (saline infusion) infusion alone. Instead, in subjects with T2DM the insulin secretion did not increase when compared with GLP-1 infusion alone, but there was an increase when compared with GIP and placebo administration, emphasizing the reduced insulinotropic effect of GIP in patients with diabetes. Moreover, in this study, energy expenditure (measured with indirect calorimetry), appetite rating, and the desire to eat were evaluated. Healthy subjects reported a higher hunger score during GIP infusion, only when compared with the placebo, whereas patients with diabetes, during co-infusion, reported a stronger desire to eat. GIP infusion determined a reduction in energy expenditure in the healthy, but not in subjects with T2DM. The limit of this study was the poor simple size, particularly in regards to the results on food intake and appetite.

In another study, the authors evaluated in two protocols “early” (0–20 min) and “late” (20–120 min) phase insulin and C-peptide responses to GLP-1 and GIP stimulation, in patients with T2DMmatching healthy subjects. GLP-1increased during the “late phase” insulin secretion to levels similar to those observed in healthy subjects, but this result was not observed with GIP. Then, in subjects with diabetes, a lack of GIP amplification of the late phase insulin response to glucose was observed [117].

In another study, 12 fasting patients with T2DMwere included; they had a mean HbA1c of 7.3% and a mean BMI of 30 kg/m^2^. All participants received an infusion of human synthetic GIP (4 pmol/kg/min) and GLP-1 (1.2 pmol/kg/min) for 6 h. Blood glucose, plasma insulin, C-peptide, glucagon, GIP, GLP-1, and free fatty acids (FFA) were determined during the experiment. In this randomized-controlled trial, GIP was unable to increase the insulinotropic effect of GLP-1 during co-infusion. In fact, comparing GLP-1 alone administration, the co-infusion resulted in similar blood glucose and insulin secretion rates in type 2 diabetes, but the suppression of plasma glucagon by GLP-1 was antagonized by GIP [118].

In conclusion, from the data provided by the GIP and GLP-1 co-infusion studies in humans, it is evident that the short-term administration of both hormones acutely increased insulin secretion and decreased blood glucose levels in healthy subjects, but not in diabetic, if compared with single GLP-1 infusion.

The lack of insulinotropic effects of GIP in type 2 diabetes, as seen in acute infusion studies, could depend on hyperglycemia. In fact, long-term glycemic control can improve the effectiveness of insulinotropic, but chronic treatment with GIP agonists alone has not been tested in humans [118,119].

Moreover, human studies evidenced that the GIP receptors was down regulated in adipose tissue in insulin-resistant states, but acute infusion of GIP under hyperinsulinism and hyperglycemia conditions promoted adipose tissue glucose uptake and triglyceride hydrolysis [74,104,120,121].

## 6. Twincretin

Considering that the co-infusion of GLP-1 and GIP receptor agonists showed an additive effect on glucose and bodyweight control, recent studies on GLP-1R/GIPR dual agonists have been started. This unimolecular co-agonist is named “twincretin”.

In study of Finan et al., (2013) [90], unimolecular dual incretin was derived from an intermixed sequence of GLP-1 and GIP and administered into rodent models of obesity and diabetes and compared with placebo or equimolar doses of exendin-4. After one week of treatment, the co-agonist improved insulin sensitivity, corrected hyperglycemia, and lowered body weight. The half-life of this molecule was prolonged either by acylation with a 16-carbon acyl chain or by attaching to a polyethylene glycol (PEG). Both versions of the co-agonists administered into *db/db* mice, ZDF rats, and into primates (cynomolgus monkeys) and demonstrated that a reduced body weight and lowered fasting glycemic in three weeks of treatment. In monkeys, dual agonist was compared with liraglutide. Instead, in healthy humans the co-agonist compared with the placebo during OGTT and showed amelioration of insulin levels and glucose tolerance. Then, GLP-1R/GIPR dual agonist corrected adiposity-induced insulin resistance and pancreatic insulin deficiency [12,92].

The beneficial effect of GLP-1R/GIPR dual agonist was confirmed in clinical trials. NNC0090-2746 (developed by Novo Nordisk) is a fatty-acylated GIP/GLP-1 dual agonist; in Phase 1 trials it achieved steady-state concentration within one week by daily dosing [122]. Once-daily doses of up to 2 mg were well tolerated [123]. After two weeks of treatment at doses > 0.75 mg, HbA1c, fasting, and postprandial plasma glucose decreased [122]. NCT02205528 was a Phase 2 clinical trial of NNC0090-2746, and was designed as a multicenter, randomized, double-blind, parallel group, placebo-controlled trial with open-label comparison, performed in patients with T2DM inadequately controlled with metformin (108 participants, 12 withdrew prematurely from the trial, 96 completed). The trial was performed in 12-weeks, investigators administered 1.8 mg of NNC 0090-2746, compared with 1.8 mg of liraglutide. Patients had a mean age of 54.8 years, the mean duration of diabetes was 8.0 years, the mean HbA1c was 8.3%, and the mean body mass index was 33.0 kg/m2. It showed improving glycated hemoglobin (−0.96% vs. placebo) and reducing bodyweight (−1.67% vs. placebo) in patients with type 2 diabetes. NNC0090-2746 reduced HbA1c similarly to the group treated with liraglutide, whereas the bodyweight reduction was significantly greater than that of liraglutide (−1.17% vs. placebo). Treatment with NNC0090-2746 was generally safe and well tolerated [18].

A 26-week treatment of GLP-1R/GIPR dual agonist, LY3298176 (Eli Lilly and Company; Indianapolis, IN, USA), was also tested. This trial was a double-blind, randomized, Phase 2 study, performed in patients with type 2 diabetes, received either once-weekly subcutaneous LY3298176 (1 mg, 5 mg, 10 mg, or 15 mg), dulaglutide (1,5 mg), or placebo [19]. Analysis included 316 participants. At baseline, mean HbA_1c_ was 8.1%, BMI was 32.6 kg/m^2^, and age was 57 years, the mean duration of diabetes was 9.0 years. LY3298176 significantly reduced HbA1c in a dose-dependent manner (1 mg, −1.06%; 5 mg, −1.73%; 10 mg, −1.89%; 15 mg, −1.94%) when compared with 1.5 mg of GLP-1R dulaglutide (−1.21%) and placebo (−0.06%), reduced bodyweight (1 mg, −1.96 kg; 5 mg, −4.62 kg; 10 mg, −6.88 kg; 15 mg, −8.67 kg) when compared with dulaglutide (−2.48 kg) and placebo (−0.40 kg) [18]. There were no reports of severe hypoglycemia. Adverse effects were similar to dulaglutide, and above all, regarding gastrointestinal adverse events, that was dose-related (23·1% for 1 mg LY3298176, 32·7% for 5 mg LY3298176, 51·0% for 10 mg LY3298176, and 66·0% for 15 mg LY3298176, 42·6% for dulaglutide, 9·8% for placebo [19].

## 7. Tirzepatide, a Dual GIP/GLP-1 Receptor Agonist: Efficacy in Phase 3 Clinical Trials

Based on promising results from Phase 1 and 2 clinical trials, a Phase 3 clinical trial program of the dual GIP/GLP-1 RA, tirzepatide (LY3298176, Eli Lilly and Company, Indianapolis, IN), was started. Tirzepatide was a 39-amino acid linear synthetic peptide that shared19 amino acids with human GIP, conjugated to a C20 fatty diacid moiety via a linker connected to the lysine residue at position 20. Due to the acylation technology, the half-life was prolonged and a once-weekly dosing regimen in humans was completed. Tirzepatide is a single molecule with agonist activity at both the GIP receptor (GIPR) and GLP-1R [124].

To date, the SURPASS program includes nine ongoing randomized controlled trials in patients with type2 diabetes: SURPASS 1 in monotherapy, SURPASS 2 versus semaglutide, SURPASS 3 versus degludec, SURPASS 4 versus glargine and established CV disease, SURPSS 5 basal insulin add-on, SURPASS AP- Combo versus glargine, and SURPASS-CVOT.

SURPASS-1was a 40-week, double-blind, randomized, Phase 3 trial. Participants (705) were subjects with T2DMinadequately controlled by diet and exercise alone and assigned to receive once a week tirzepatide (5, 10, or 15 mg), or a placebo. The mean baseline of HbA_1c_ was 7.9%, of age was 54.1 years (SD 11.9), diabetes duration was 4.7 years, and of body-mass index was 31.9 kg/m^2^. At 40 weeks, tirzepatide induced a dose-dependent decreasing of HbA1c and bodyweight. Gastrointestinal events were the most frequent adverse effects, such as nausea (12–18% vs. 6%), diarrhea (12–14% vs. 8%), and vomiting (2–6% vs. 2%). Severe hypoglycemia was not reported [125].

SURPASS-2 was an open-label, 40-week, Phase 3 clinical trial.1879 patients enrolled and received tirzepatide at a dose of 5 mg, 10 mg, or 15 mg, or semaglutide at a dose of 1 mg. At baseline, the mean HbA1c was 8.28%, the mean age was 56.6 years, and the mean weight was 93.7 kg. Tirzepatide at all doses was non inferior and superior to semaglutide as regards to the mean change in theHbA1c; instead reductions in body weight were greater with Tirzepatide than with semaglutide (*p* < 0.001). The most common adverse events were gastrointestinal [126].

In another Phase 3 study, there were 1947 subjects enrolled with T2DM; at baseline mean HbA_1c_ of 8.17% (SD 0.91) and a mean weight of 94.3 kg (SD 20.1). Researchers have evaluated the efficacy and safety of tirzepatide versus titrated insulin degludec in subjects with T2DMinadequately controlled by metformin, with or without SGLT2 inhibitors. The SURPASS-3 findings showed that people with T2DM can obtain a better glycemic control with tirzepatide than with insulin degludec, while losing weight rather than gaining weight. A higher incidence of gastrointestinal adverse effects was reported in participants treated with tirzepatide, compared with those treated with degludec [127].

SURPASS-4 was an open-label global trial comparing the safety and efficacy of tirzepatide with titrated insulin glargine in 2002 people with T2DMwith an increased CV risk who were treated with metformin, a sulfonylurea, or an SGLT-2 inhibitor. Study participants had a mean duration of diabetes of 11.8 years, a baseline HbA1c of 8.52%, and a baseline weight of 90.3 kg. More than 85% of participants had a history of cardiovascular diseases. The highest dose of tirzepatide led to a significant HbA1c reduction at 52 weeks [128].

SURPASS-5 was designed for comparing tirzepatide versus the placebo as an add-on therapy to titrated insulin glargine. In this randomized, Phase 3, double-blind trial, 475 participants enrolled. Tirzepatide was associated with greater HbA1c reductions and body weight reductions than placebo therapy. At 40 weeks, tirzepatide led to a reduction from baseline of HbA1c of 2.59%with the highest dose and reduced body weight by 6.2 kg, 8.2 kg, and 10.9 kg with the three respective doses of active drug [129].

And finally, SURPASS AP-Combo and SURPASS-CVOT are ongoing.

SURPASS AP-Combo testing tirzepatide versus insulin glargine, over a 40-week period, in people taking metformin with or without a sulfonylurea of at least half the maximum dose (NCT04093752, estimated study completion in March 2022).

In SURPASS-CVOT tirzepatide is up against dulaglutide 1.5mg, which has a confirmed cardioprotective effect. The investigators are assessing a three-point major adverse cardiovascular event endpoint (myocardial infarction, stroke, and cardiovascular death), over an estimated maximum of 54months (NCT04255433, estimated study completion in October 2024).

The results of the major studies and other informationof SURPASS Program are summarized in Table 1.

SURPASS J-mono (NCT03861052) and SURPASS J-combo (NCT03861039) are ongoing clinical trials for the Japanese market; the first is comparing weekly tirzepatide (5, 10, or 15 mg) against weekly dulaglutide 0.75 mg in Japanese people taking no other glucose-lowering medications during the trial, and the primary endpoint is a change in HbA1c, and the second is a safety study that will monitor adverse events in Japanese people given tirzepatide (5, 10, or 15 mg) in addition to non-incretin-based antidiabetes medications over 52 weeks of treatment.

SURMOUNT-1 trial (NCT04184622, estimated study completion in May 2024) is testing the ability of tirzepatide (at doses of 5, 10, or 15 mg) to reduce weight in subjects with diabetes and obesity at 72 weeks.

Combination therapies allow for intervention on the multiple mechanisms responsible for hyperglycemia and could reduce residual CV risk in diabetic patients in the future [130,131,132]. Moreover, due to the increased incidence of T2DM and its poor prognosis, early and intensive treatment is important [133].

## 8. Conclusions

In the last years, scientific research has focused its interest on the synergistic action of GLP-1 and GIP. In vitro and in vivo preclinical studies have demonstrated that co-infusion of GLP-1 and GIP leads to improvement of glucose control and bodyweight regulation, compared with separate administration of each hormone, and these findings have been confirmed in human studies.

Based on promising preclinical data, a dual GIP/GLP-1 receptor agonist, named “twincretin”, has been developed and emerging evidence showed that in Phase 3 clinical trials, tirzepatide was able to induce a potent glucose-lowering and weight loss with adverse effects comparable to those of established GLP-1 receptor agonist. The long-term and cardiovascular outcomes of tirzepatide will be investigated in the SURPASS-CVOT.

Furthermore, recent preclinical studies have demonstrated beneficial effects when GLP-1 R was combined with glucagon receptor agonists. Indeed, GLP-1R has been matched with Glucagon receptor agonist in unimolecular agonist (SAR425899, Sanofi, Paris, France) and preclinical studies showed that decreased bodyweight in diet-induced obese mice and decreased glucose levels in db/db mice [134].

Similarly, another GLP-1R/GR dual agonist (MEDI0382, MedImmune, Gaithersburg, MD, USA), was able to significantly reduce bodyweight and to ameliorate glucose levels when compared with liraglutide, in non-human primates [135].

Based on these evidences, GLP-1R/GIPR/GR triple agonists have been under development and in preclinical studies have shown that the reduction of bodyweight was greater than the same dose of a GLP-1/GIPR dual agonist in diet-induced obese mice [104,136].

## Figures and Tables

**Table 1:** Randomized controlled trials of tirzepatide in patients with T2DM.

Study	Population	Sample Size	Intervention	Primary Outcome	Results
**SURPASS-1 (2021)**	T2DM inadequately controlled with diet and exercise alone	478 tirzepatide 5 mg (n = 121); tirzepatide 10 mg (n = 121); tirzepatide 15 mg (n = 121); placebo (n = 115)	Tirzepatide (5, 10, or 15 mg), or placebo	To assess efficacy, safety, and tolerability oftirzepatide versus placebo, and change from baseline in HbA1c timeframe: baseline week 40	At 40 weeks, tirzepatide induced a dose-dependent decreasing of HbA1c (mean HbA_1c_ decreased from baseline by 1.87% with tirzepatide 5 mg, 1.89% with tirzepatide 10 mg, and 2.07% with tirzepatide 15 mg versus +0·04% with placebo), and bodyweight loss ranging from 7.0 to 9.5 kg.
**SURPASS-2 (2021)**	Patients with T2DM treated with unchanged dose of metformin > 1500 mg/day for at least 3 months prior to screening	1879 Patients are randomized in a 1:1:1:1 ratio, to receive tirzepatide at a dose of 5 mg, 10 mg, or 15 mg, or semaglutide at a dose of 1 mg.	Tirzepatide (5 mg, 10 mg, 15 mg) versus semaglutide once weekly as add-on therapy to metformin	To compare the effect of the tirzepatide to semaglutide onchange from baseline in HbA1c (10 mg and 15 mg) at 40 week.	Tirzepatide at all doses was noninferior and superior to semaglutide as regards t othe mean change in theHbA1c; instead reductions in body weight were greater with tirzepatide than with semaglutide (*p* < 0.001).
**SURPASS-3 (2021)**	Subjects with T2DM inadequately controlled by metformin with or without SGLT2 inhibitors	1947	Tirzepatide (one weekly, 5, 10, or 15 mg) versus titrated insulin degludec add-on metformin with or without SGLT2 inhibitors	Change from baseline in HbA1c at 52 week.	People with T2DMobtained better glycemic control with tirzepatide than insulin degludec, while losing rather than gaining weight.
**SURPASS-4 (2021)**	People with T2DM with increased CV risk who are treated with metformin, or a sulfonylurea or an SGLT-2 inhibitor	2002	Tirzepatide (5 mg, 10 mg, and 15 mg) with titrated insulin glargine	Change from baseline in HbA1c (10 mg and 15 mg) at 52 weeks, and to assess the efficacy and safety of tirzepatide taken once a week to insulin glargine taken once daily in participants with T2DMand increased cardiovascular risk.	The highest dose of tirzepatide led to an HbA1c reduction of 2.58% and reduced body weight by −11.7 kg compared with insulin glargine at 52 weeks.
**SURPASS 5 (2021)**	Patients with T2DM inadequately controlled on insulin glargine with or without metformin	475	Tirzepatide versus placebo in patients with T2D Minadequately controlled on insulin glargine with or without metformin	To evaluate the safety and efficacy of tirzepatide to placebo in participants with T2DMthat are already on insulin glargine, with or without metformin, and change from baseline in HbA1c (10 mg and 15 mg) at 40 weeks	Tirzepatide was associated with greater HbA1c reductions and body weight reductions than in placebo therapy. Additionally, results indicated 97% of participants receiving tirzepatide achieved an HbA1c of less than 7% and 62% achieved an HbA1c of less than 5.7%.
**SURPASS-6** **(recruiting)**	T2DM inadequately controlled on insulin glargine (U100) with or without metformin	1182	Tirzepatide once weekly versus insulin lispro (U100) three times daily	To compare the safety and efficacy of the tirzepatide to insulin lispro (U100) three times a day in participants with T2DM that are already on insulin glargine (U100), with or without metformin, monitoring change from baseline in HbA1c	Estimated study completion date: 18 November 2022
**SURPASS-CVOT** **(Recruiting)**	Patients with T2DM and increased cardiovascular risk	12,500	Tirzepatide (5 mg, 10 mg, 15 mg) versus dulaglutide (1.5 mg)	Time to first occurrence of death from cardiovascular (CV) causes, myocardial infarction (MI), or stroke (MACE-3)	Estimated study completion date: 17 October 2024
**SURPASS AP-Combo** **(Active, not recruiting)**	Subjects with T2DM treated with metformin with or without a sulfonylurea	917	Tirzepatide (5 mg, 10 mg, 15 mg) once weekly versus titrated insulin glargine add-on metformin with or without a sulfonylurea	Mean change from baseline in HbA1c (10 mg and 15 mg)	Estimated study completion date: 26 November 2021

## Abbreviations

BMI	Body mass index
CV	Cardiovascular
cAMP	Cyclic-adenosine-monophosphate
CVOT	Cardiovascular outcome trial
DPP-4	Dipeptidyl peptidase 4
FDA	United States Food and Drug Administration
FFA	Free fatty acids
GIP	Glucose-dependent insulinotropic polypeptide
GIPRs	GIP receptors
GLP-1	Glucagon-like peptide-1
GLP-1 R	Glucagon-like peptide-1 receptor
GLP-1 RA	Glucagon-like peptide-1 receptor agonists
GR	Glucagon receptor
HbA1c	Glycated hemoglobin A1c
i.v.	Intravenous
MACE	Major cardiovascular events
MI	Myocardial infarction
OGTT	Oral glucose tolerance test
PEG	Polyethylene glycol
SGLT	Sodium-glucose co-transporter type
SD	Standard deviation
T2DM	Type 2 diabetes mellitus
